# Molecular dynamics simulations reveal differences in the conformational stability of FtsZs derived from *Staphylococcus aureus* and *Bacillus subtilis*

**DOI:** 10.1038/s41598-024-66763-x

**Published:** 2024-07-11

**Authors:** Taichi Takasawa, Takashi Matsui, Go Watanabe, Yoshio Kodera

**Affiliations:** 1 Department of Physics, School of Science, Kitasato University, 1-15-1 Kitasato, Minami-Ku, Sagamihara, Kanagawa 252-0373 Japan; 2 Center for Disease Proteomics, School of Science, Kitasato University, 1-15-1 Kitasato, Minami-Ku, Sagamihara, Kanagawa 252-0373 Japan; 3 Department of Data Science, School of Frontier Engineering, Kitasato University, 1-15-1 Kitasato, Minami-Ku, Sagamihara, Kanagawa 252-0373 Japan; 4 Kanagawa Institute of Industrial Science and Technology (KISTEC), 705-1 Shimoimaizumi, Ebina, Kanagawa 243-0435 Japan

**Keywords:** Computational biophysics, Molecular conformation, Bacterial structural biology, Biomaterials - proteins

## Abstract

FtsZ is highly conserved among bacteria and plays an essential role in bacterial cell division. The tense conformation of FtsZ bound to GTP assembles into a straight filament via head-to-tail associations, and then the upper subunit of FtsZ hydrolyzes GTP bound to the lower FtsZ subunit. The subunit with GDP bound disassembles accompanied by a conformational change in the subunit from the tense to relaxed conformation. Although crystal structures of FtsZ derived from several bacterial species have been determined, the conformational change from the relaxed to tense conformation has only been observed in *Staphylococcus aureus* FtsZ (*Sa*FtsZ). Recent cryo-electron microscopy analyses revealed the three-dimensional reconstruction of the protofilament, in which tense molecules assemble via head-to-tail associations. However, the lower resolution of the protofilament suggested that the flexibility of the FtsZ protomers between the relaxed and tense conformations caused them to form in less-strict alignments. Furthermore, this flexibility may also prevent FtsZs other than *Sa*FtsZ from crystalizing in the tense conformation, suggesting that the flexibility of bacterial FtsZs differs. In this study, molecular dynamics simulations were performed using *Sa*FtsZ and *Bacillus subtilis* FtsZ in several situations, which suggested that different features of the FtsZs affect their conformational stability.

## Introduction

FtsZ is highly conserved among bacteria and plays an essential role in bacterial cell division^1,2^. During cell division, the bound nucleotide in monomer FtsZ exchanges from GDP to GTP, and then FtsZ bound to GTP assembles into a straight filament via head-to-tail associations. The FtsZ filament serves as a scaffold at the cell division position for the partners and promotes further cell division processes. FtsZ is a GTPase; thus, the upper subunit of FtsZ hydrolyzes GTP accommodated in the lower subunit. Consequently, the penultimate subunit with GDP bound in the tip of the filament disassembles accompanied by a conformational change in the subunit from the tense to relaxed conformations, in contrast, the monomer FtsZ bound with GTP attaches at the other end of the filament, promoting that the filament treadmills and then the multiple filament patches move around the cell division site^3–12^.

Although the above hypothesis has been proposed, details regarding the assembly cycle machinery remain unclear, even though the crystal structures of FtsZ derived from several bacterial species have been determined^5,11,13–20^. This is because no conformational change between the tense and relaxed conformations has been observed, except in *staphylococcal* FtsZs^5,8,15,16,19,20^. A principal component analysis revealed that one of the eigenvectors described the difference in conformation between the polymerized and unpolymerized conditions^21^. However, all conformations within the subspace of the polymerized condition have been derived from *Staphylococcus* alone. Therefore, it is also unclear whether the proposed conformational changes are conserved among bacteria or associated with differences between bacteria species.

FtsZ is composed of two subdomains linked by the T6 loop, H7 helix, the T7 loop, and H8 helix. The N-terminal subdomain (NTD, residues 12–173) accommodates the nucleotide, and the C-terminal subdomain acts as a GTPase-activating domain (GAD, residues 223–310). In the conformational change from the relaxed to tense conformation observed in *Staphylococcus aureus* FtsZ (*Sa*FtsZ) and its mutant^8,15,16^, the H7 helix and T7 loop are down-shifted approximately one helical pitch, accompanied by a reorientation of the GAD (Supplementary Fig. S1). Consequently, the area of the interface between the upper and lower subunits made by the tense conformation in the filament is increased by 40% compared with the interface formed by the relaxed conformation, and the T7 loop is located deep within the lower subunit near the nucleotide. Recent cryoEM analyses revealed the three-dimensional reconstruction of the protofilaments derived from *Escherichia coli* FtsZ (*Ec*FtsZ), *Mycobacterium tuberculosis* FtsZ (*Mtb*FtsZ), and *Klebsiella pneumoniae* (*Kp*FtsZ)^8,21,22^, and the tense conformation of *Sa*FtsZ fit well into a density map of the protofilaments rather than the corresponding relaxed monomer of *Ec*FtsZ, *Mtb*FtsZ, or *Kp*FtsZ. These CryoEM reconstructions predicted that the conformational change induces an assembly switch upon polymerization. However, the lower resolutions of *Ec*FtsZ, *Mtb*FtsZ, and *Kp*FtsZ observed using CryoEM suggested a less strict alignment or mixture of both the tense and relaxed conformations in the straight filament as predicted in previous studies^11,23^. FtsZ may exhibit flexible conformations for the conformational change triggered by bound nucleotide and/or polymerization; thus, the difference in flexibility of the *Ec*FtsZ, *Mtb*FtsZ, and *Kp*FtsZ protomers between the relaxed and tense conformations probably caused them to not form a filament strictly. Furthermore, the flexibility may also prevent the FtsZs from crystalizing in the tense conformation.

A molecular dynamics (MD) simulation is a physical-based theoretical method for capturing the movement of each atom in a protein or other molecular system. MD simulations can help researchers understand the mechanisms of a wide variety of biological phenomena, such as structural dynamics, perturbations, and processes of biomolecular systems. Due to rapid developments in computing power, MD simulations have become much more powerful and accessible in recent years. MD simulations can now be used to investigate the atomic dynamics of protein folding and misfolding^24^, elucidate the mechanisms of membrane ion channel functions^25^, and accurately predict the recognition and association of a protein and ligand^26^. Three-dimensional protein structures can be obtained with atomic-level accuracy using artificial intelligence (AI) systems (i.e., AlphaFold2 and RoseTTaFold)^27,28^. Although AI can be used to accurately predict a protein’s structure based on crystal structure database information, it is still difficult to reproduce the structure of protein–ligand complexes, as AlphaFold2 typically generates a model in only one functional state, which is usually the inactive state^29–31^.

The present study assessed the differences in flexibility between bacterial FtsZs in several conformational states using MD simulations of the monomer FtsZ within the tense and relaxed conformations bound to GTP and GDP, respectively. Although both conformations of *Sa*FtsZ were determined by X-ray crystallography^16^, the tense conformation of *Bacillus subtilis* FtsZ (*Bs*FtsZ) has not been observed. A tense model of *Bs*FtsZ was generated from *Sa*FtsZ as a template using SWISS-MODEL^32^, and then all FtsZ structures were equilibrated in aqueous solution, and their stability and fluctuation were estimated.

## Results

### Evaluation of equilibration of the system

**Figure 1:**
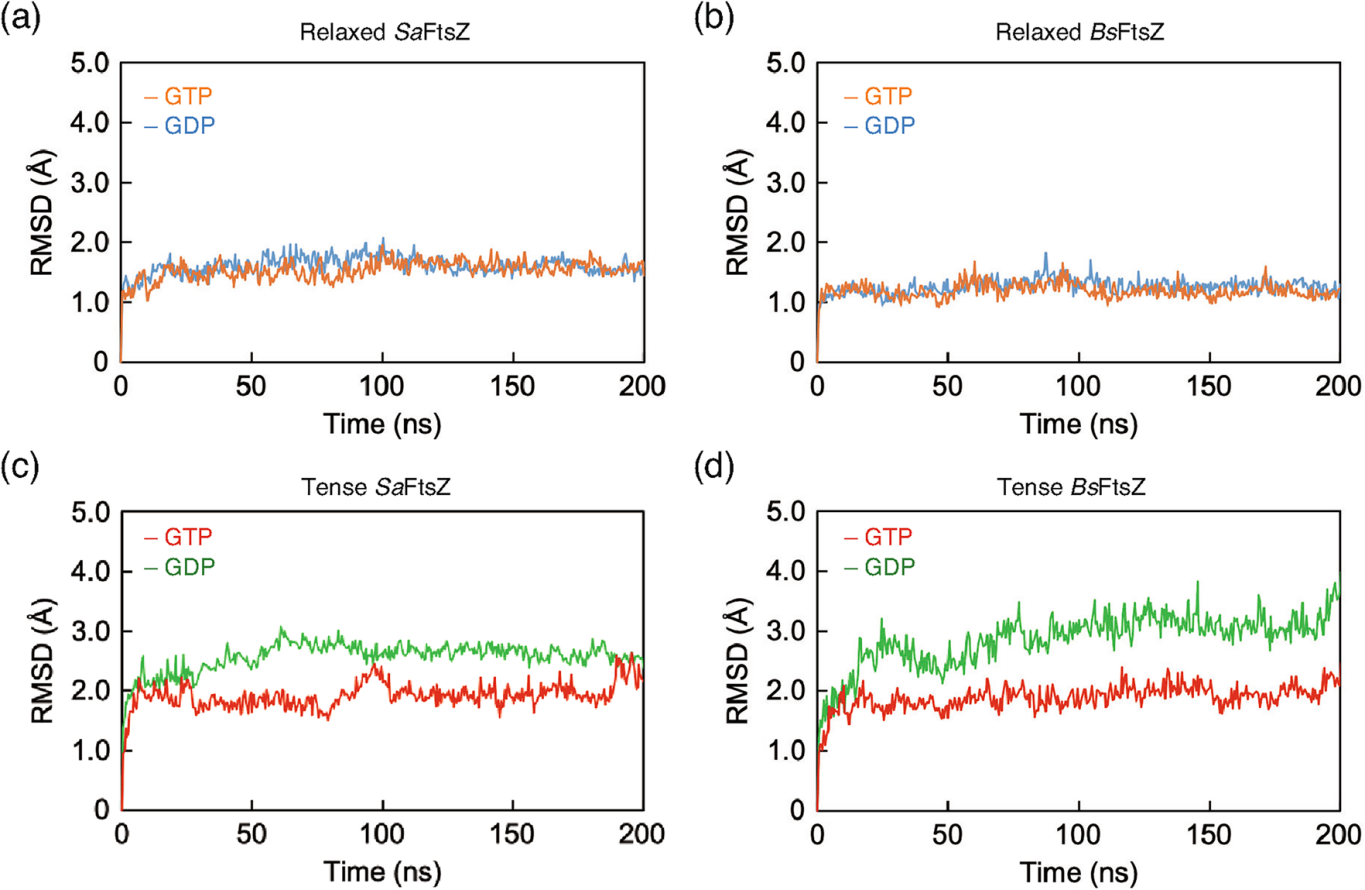
RMSD trajectories during 200-ns MD simulations. (**a**,**b**) MD simulations of relaxed molecules derived from (**a**) *Sa*FtsZ (5H5G molecule B^16^) and (**b**) *Bs*FtsZ (2VXY^14^)^[]^, respectively. Relaxed conformations bound to GTP and GDP are drawn as orange and blue lines, respectively. (**c**,**d**) MD simulations of tense molecules derived from (**c**) *Sa*FtsZ (5H5G molecule A^16^) and (**d**) SWISS-MODEL of *Bs*FtsZ templated from 5H5G molecule A^16^. Tense conformations bound to GTP and GDP are depicted as red and green lines, respectively.

All systems were equilibrated during 200-ns MD simulations (Fig. 1). In the relaxed conformation of *Sa*FtsZ, the root mean square deviation (RMSD) values of *Sa*FtsZ bound to both GTP and GDP were stable, at 1.5 ± 0.1 Å and 1.6 ± 0.1 Å after 50 ns, respectively (Fig. 1a). These systems equilibrated rapidly. Rapid equilibration was also observed in the relaxed conformation of *Bs*FtsZ bound to GTP and GDP, with RMSD values of 1.2 ± 0.1 Å and 1.3 ± 0.1 Å after 50 ns, respectively (Fig. 1b). In contrast to the relaxed conformation, the tense conformation of *Sa*FtsZ showed a different equilibration pattern in binding of GTP and GDP (Fig. 1c). For the *Sa*FtsZ tense conformation bound to GTP (tense *Sa*FtsZ-GTP), the RMSD value reached ~ 1.5 Å at 20 ns and remained stable for the remainder of the simulation. The RMSD value of the tense conformation bound to GDP (tense *Sa*FtsZ-GDP) gradually reached a plateau, with an average value during the equilibration of 2.7 Å. The tense conformation of *Bs*FtsZ exhibited a similar pattern (Fig. 1d). Tense *Bs*FtsZ showed different trajectories in binding GTP versus GDP, with average RMSD values for these two systems of 1.9 ± 0.1 Å over the period of 10–200 ns and 3.1 ± 0.1 Å over the period of 70–200 ns, respectively. These results suggest that the relaxed conformation in the crystal structure was already thermodynamically stable, regardless of bacterial species and bound nucleotide. In contrast, the tense conformations of both FtsZs may form a higher energetic state. Furthermore, the tense conformation of FtsZ-GDPs derived from both species exhibited a change in the stable conformation of approximately 3 Å-difference relative to the initial state during the 200-ns MD simulations.

### Flexibility of the nucleotide-bound formations

The average structure during the last 50 ns of the equilibrated state was generated, and the flexibility of each residue during that period was evaluated based on the root mean square fluctuation (RMSF) value (Supplementary Figs. S2–S3). Several specific regions fluctuated in relaxed *Sa*FtsZ bound to either GTP or GDP (Supplementary Fig. S2a). Higher fluctuations occurred in the H3 helix (Ser85–Asp97) in the NTD and H8 helix (Asp209–Ser219) in the linker region of relaxed *Sa*FtsZ-GDP. In contrast, T6 (Asp174–Ala182) and T7 (Ser204–Leu207) in the linker region, and T10 (Gly267–Leu270) and T11 (Asn299–Glu305) in the GAD exhibited greater flexibility when binding GTP (Supplementary Fig. S2a,b). Although the differences in flexibility between bound nucleotides in tense *Sa*FtsZs were lower than those in relaxed *Sa*FtsZs (Supplementary Fig. S1a,c), several residues in tense *Sa*FtsZ also exhibited different dynamics depending on the bound nucleotide. The RMSF value of Ser204 at the T7 loop (Leu201–Leu209) in tense *Sa*FtsZ-GTP was 3.8 times higher than that in *Sa*FtsZ-GDP. In contrast, the RMSF value of Asn220 at the T8 loop (Asn220–Ser223) in tense *Sa*FtsZ-GTP was 3.7 times lower than that of *Sa*FtsZ-GDP (Supplementary Fig. S2c,d).

Loops T6 and T7, which are the flanks of the H7 helix, showed higher deviations in relaxed and tense *Sa*FtsZ-GTP compared with the GDP-bound states. Ser204 of the T7 loop in tense *Sa*FtsZ-GTP exhibited a higher RMSF value of 3.3 Å compared with the relaxed state value of 1.4 Å. The RMSF values of Gly33 at H1 helix (Gly20-HIS33) and Asn35 at the T1 loop (Gly34–Glu39) in tense *Sa*FtsZ-GTP were > 2.0 Å; Ser269 in the T10 loop was only observed in the relaxed conformation and exhibited an RMSF value of 2.7 Å.

A comparison of the relaxed conformations of *Sa*FtsZ and *Bs*FtsZ indicated that relaxed *Bs*FtsZ bound to either nucleotide was more stable than those of *Sa*FtsZ (Supplementary Fig. S2, S3). Only Lys175 in the T6 loop markedly fluctuated in relaxed *Bs*FtsZ bound to GDP and GTP, with RMSF values of 2.2 Å and 1.5 Å, respectively. However, the RMSF value of Lys175 in the T6 loop of relaxed *Sa*FtsZ-GDP was lower than that of the molecule bound to GTP (1.5 versus 2.0 Å, respectively). Furthermore, relaxed *Sa*FtsZ exhibited several fluctuating loops, including the T6 loop, and these loops showed higher deviations in the GTP-bound state, whereas no similar behavior was exhibited by relaxed *Bs*FtsZ-GTP.

A comparison of the tense conformations of *Sa*FtsZ and *Bs*FtsZ showed that *Bs*FtsZ formed a more stable conformation than *Sa*FtsZ under the same bound nucleotide condition. The T1 and T7 loops in tense *Sa*FtsZ-GTP were flexible, whereas no marked fluctuation in those loops was observed in tense *Bs*FtsZ-GTP. However, the RMSF value of Lys175 of the T6 loop in tense *Bs*FtsZ-GTP, at 2.6 Å, was similar to the values of that in the tense *Sa*FtsZ bound to GTP and GDP, at 2.8 Å and 2.4 Å, respectively. A significantly large fluctuation at Glu139 of the T5 loop (Arg133–Arg141) was found only in tense *Bs*FtsZ bound to GDP and GTP, with RMSF values of 2.2 Å and 3.5 Å, respectively.

### Structural comparisons of MD simulation models with reference structures

**Figure 2:**
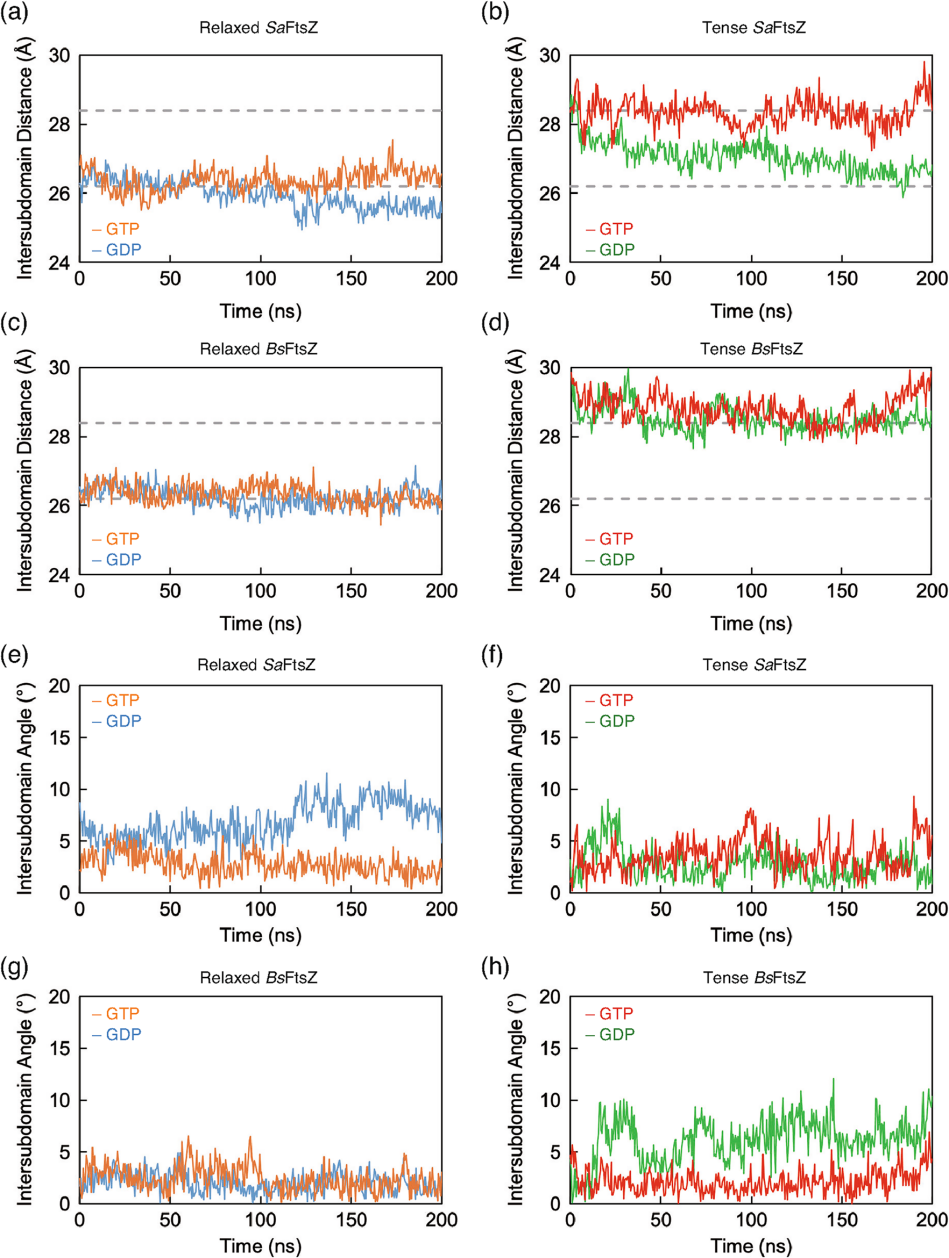
Trajectories of 200-ns MD simulation inter-subdomain reorientation. (**a**,**b**) Inter-subdomain distances of (**a**) relaxed and (**b**) tense conformations of *Sa*FtsZ. (**c**,**d**) Inter-subdomain distances of (**c**) relaxed and (**d**) tense conformations of *Bs*FtsZ. Inter-subdomain distances derived from tense (PDB 5H5G mol A^16^) and relaxed (PDB 5H5G mol B^16^) forms are shown as dotted lines, with values of 28.4 and 26.2 Å, respectively. (**e**,**f**) Inter-subdomain angles of (**e**) relaxed and (**f**) tense conformations of *Sa*FtsZ. (**g**,**h**) Inter-subdomain angles of (**g**) relaxed and (**h**) tense conformations of *Bs*FtsZ. Relaxed conformations bound to GTP and GDP are drawn as orange and blue lines, respectively. Tense conformations bound to GTP and GDP are depicted as red and green lines, respectively.

To better understand the conformational differences, MD-simulated structures were compared with reference structures. First, the structural features of the tense and relaxed conformations against the crystal structures deposited previously in the PDB database^5,14,16,17,33^ were identified based on inter-subdomain distances using the relative distance between the centers in each subdomain (Supplementary Table S2). The inter-subdomain distances between the centers were 28.4 ± 0.1 Å and 26.2 ± 0.5 Å in the tense and relaxed conformations, respectively. Figure 2 shows the trajectories in each conformation of *Sa*FtsZ and *Bs*FtsZ. The relaxed conformations of both bacterial FtsZs bound to GDP and GTP remained at a stable distance, as observed in the crystal structures of the relaxed FtsZs, at 26.0 ± 0.4 Å and 26.4 ± 0.3 Å in *Sa*FtsZ and at 26.2 ± 0.3 Å and 26.3 ± 0.3 Å in *Bs*FtsZ, respectively. The inter-subdomain distance of tense *Bs*FtsZ, regardless of bound nucleotide, exhibited an average value of approximately 28 Å. In contrast to tense *Bs*FtsZ, although tense *Sa*FtsZ-GTP exhibited a stable average distance similar to that calculated from the tense conformation in the crystal structure, tense *Sa*FtsZ-GDP exhibited a shift in the distance, which was similar to the average value of the relaxed conformation, at 26.6 ± 0.3 Å for the last 50 ns. These data suggested that both FtsZs assumed a different conformation accompanied by a difference in stability, especially in the tense conformation bound to GDP.

**Figure 3:**
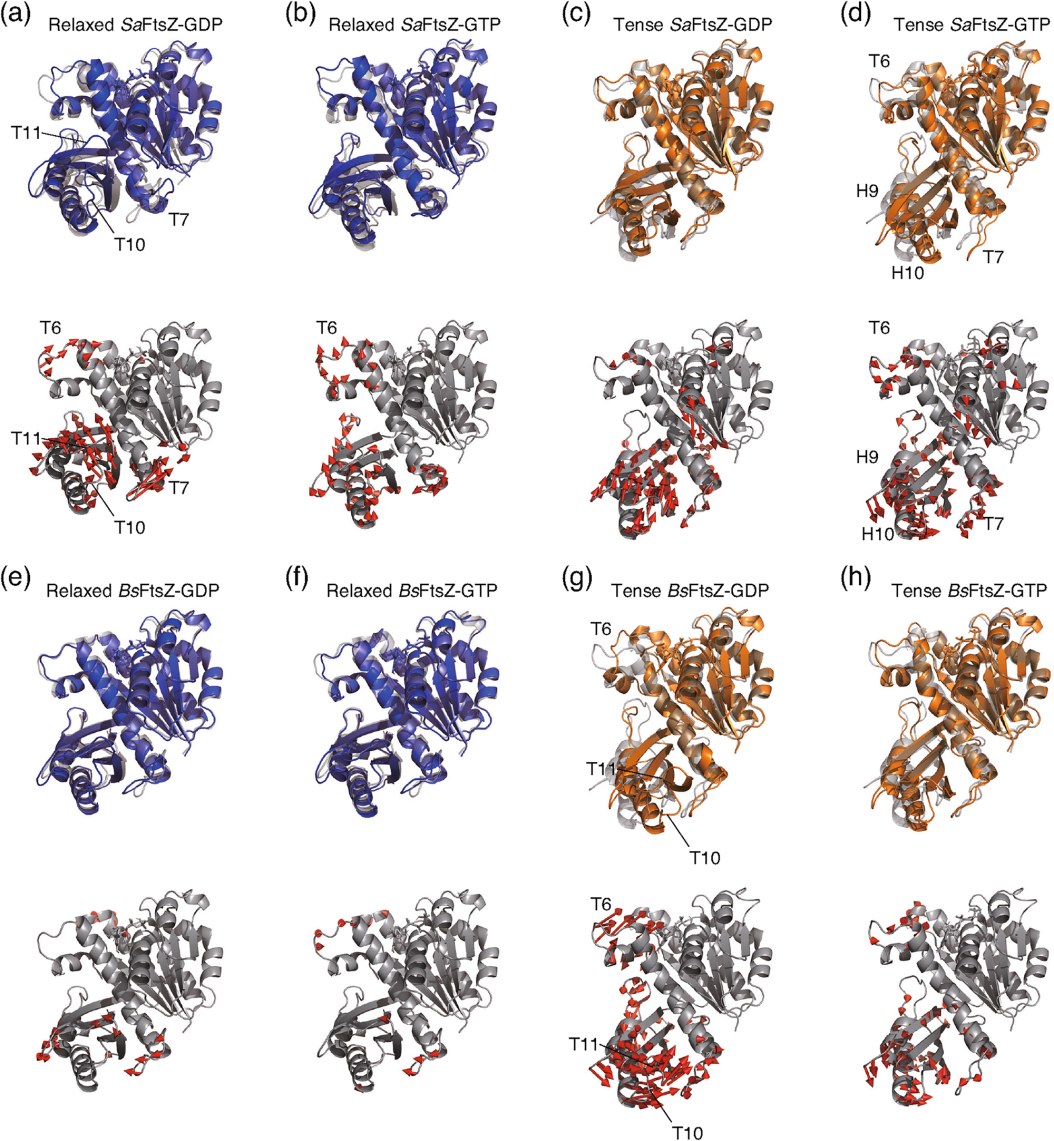
Average structure during last 50 ns of MD simulations compared with the reference structure. Average structure of (**a**) relaxed *Sa*FtsZ bound to GDP compared with the crystal structure (PDB 2VXY^14^, *Bs*FtsZ). (**b**) Relaxed *Sa*FtsZ bound to GTP compared with the crystal structure (PDB 5H5G mol B^16^). Tense *Sa*FtsZ bound to (**c**) GDP and (**d**) GTP compared with the crystal structure (PDB 5H5G mol A^16^). Relaxed *Bs*FtsZ bound to (**e**) GDP and (**f**) GTP compared with the crystal structure (PDB 2VXY^14^). Tense *Bs*FtsZ bound to (**g**) GDP and (**h**) GTP compared with the SWISS-MODEL structure. The average structures of MD simulations started from the relaxed and tense forms, and the reference structures are depicted as blue, orange, and gray cartoons, respectively. Conformational changes from the crystal or model structure to the average structures indicated in panels (**a**) through (**h**) are illustrated on the initial structure (gray color) as vectors. Coordinate differences > 2.0 Å are drawn as red arrows.

In contrast to the inter-subdomain distances, no rotational axis in all average structures against the corresponding reference structure was detected using DynDom^34^, suggesting that the relative orientation between subdomains as seen in the difference between relaxed and tense conformations in the previous investigation^5,8,16,19,20^ was not observed. Furthermore, the conformations of NTD and GAD in the average structures during the last 50-ns simulations were superimposed well with those in the crystal structures, respectively (Fig. 3 and Supplementary Fig. S4), suggesting that there was no conformational change in each subdomain. However, the whole conformations of all simulated structures seemed to be changed (Supplementary movies S1–S8). Then, the vector angles between the GAD and NTD of the MD simulations were also evaluated against the reference conformation (Fig. 2e–h, Supplementary Tables S3–S4). In the 200-ns MD simulation, relaxed *Sa*FtsZ-GTP and tense *Sa*FtsZ bound to both nucleotides exhibited lower relative vector angles compared with the initial conformations, at 2.8 ± 1.1°, 2.7 ± 1.6°, and 3.6 ± 1.6°, respectively (Supplementary Table S3). In contrast to those conformations, the relative vector angle of relaxed *Sa*FtsZ-GDP compared with the initial model (PDB 5H5G, mol B^16^) was 6.8 ± 1.8°. However, the vector between the subdomains in relaxed *Sa*FtsZ-GDP formed angles with the subdomains of *Bs*FtsZ (PDB 2VXY^14^) and *Methanocaldococcus jannaschii* FtsZ (*Mj*FtsZ) (PDB 1W59^33^) of 2.0 ± 1.0° and 2.1 ± 1.0°, respectively (Supplementary Table S3), suggesting that in the MD simulation, relaxed *Sa*FtsZ-GDP equilibrated to assume a similar conformation to the crystal structures of *Bs*FtsZ and *Mj*FtsZ.

The relaxed *Bs*FtsZ-GDP, relaxed *Bs*FtsZ-GTP, and tense *Bs*FtsZ-GTP exhibited lower vector angles compared with the references, at 2.0 ± 1.0°, 2.4 ± 1.2°, and 2.1 ± 1.2°, respectively (Supplementary Table S4). Only tense *Bs*FtsZ-GDP exhibited a vector angle > 5.7 ± 2.1° during the entire MD simulation. Thus, only tense *Bs*FtsZ-GDP assumed a conformation with a relative orientation much different from that of previous structures in the PDB.

The conformational changes relative to the reference structures are summarized in Fig. 3 and Supplementary Fig. S5. In the average conformation during the last 50 ns of the MD simulation, the conformation of relaxed *Sa*FtsZ-GDP was similar to that of *Bs*FtsZ (PDB 2VXY^14^), except for slight conformational differences in the T10 and T11 loops in the GAD and in the T7 loop (Fig. 3a). No significant conformational changes were observed between relaxed *Sa*FtsZ-GTP and the reference structure (SaFtsZ, PDB 5H5G mol A^16^), even though there was a small difference in the rotational angle of 2.8° between both conformations over 2-Å conformational shifts at positions far from the NTD (Fig. 3b and Supplementary Table S3).

**Figure 4:**
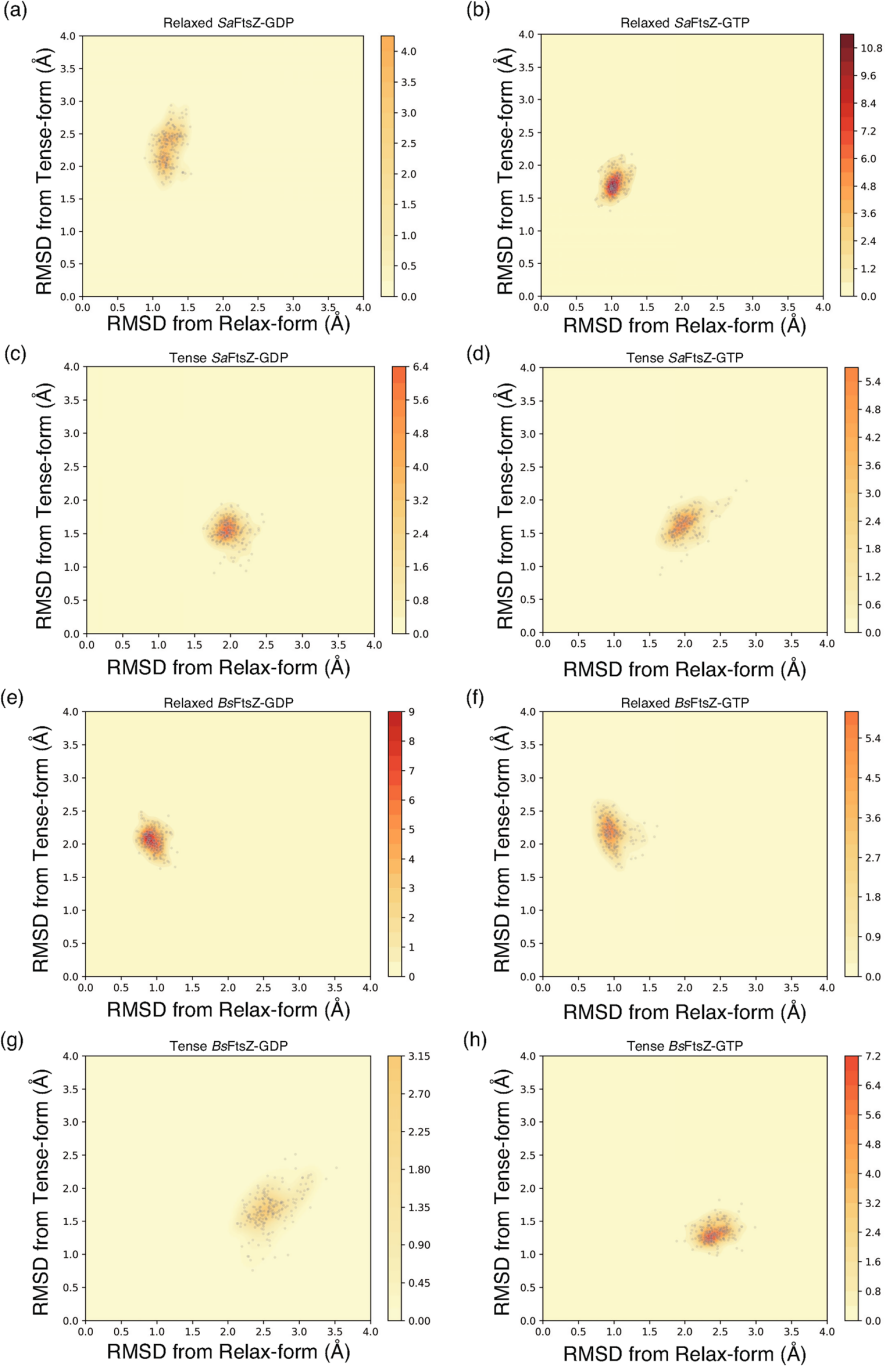
Differences in RMSD values of MD simulations with the crystal structure. Trajectories of (**a**) *Sa*FtsZ relaxed-GDP compared with the relaxed-form (PDB 2VXY^14^) and tense-form (PDB 5H5G mol A^16^). (**b**) *Sa*FtsZ relaxed-GTP compared with relaxed-form (PDB 5H5G mol B^16^) and tense-form (PDB 5H5G mol A^16^). (**c**) *Sa*FtsZ tense-GDP compared with relaxed-form (PDB 5H5G mol B^16^) and tense-form (PDB 5H5G mol A^16^). (**d**) *Sa*FtsZ tense-GTP compared with relaxed-form (PDB 5H5G mol B^16^) and tense-form (PDB 5H5G mol A^16^). (**e**) *Bs*FtsZ relaxed-GDP compared with relaxed-form (PDB 2VXY^14^) and tense-form (PDB 5H5G mol A^16^). (**f**) *Bs*FtsZ relaxed-GTP compared with relaxed-form (PDB 2VXY) and tense-form (PDB 5H5G mol A^16^). (**g**) *Bs*FtsZ tense-GDP compared with relaxed-form (PDB 2VXY^14^) and tense-form (PDB 5H5G mol A^16^). (**h**) *Bs*FtsZ tense-GDP compared with relaxed-form (PDB 2VXY^14^) and tense-form (PDB 5H5G mol A^16^). Each 1-ns step is depicted as a gray dot. The kernel density estimation for each conformation is shown as a contour map, with the contour level of each panel also shown.

Figure 4 shows a map of the kernel density estimation, which is the trajectory of the RMSD differences for each *Sa*FtsZ conformation at each 1-ns step against the relaxed and tense forms. The MD simulation revealed that the relaxed *Sa*FtsZ-GDP exhibited a 1–1.5 Å difference with approximately 0.5-Å deviations from the reference of the relaxed conformation (Fig. 4a), suggesting that the conformation of relaxed *Sa*FtsZ-GDP fluctuates in the relaxed conformation by over 2-Å difference compared with the tense conformation. The relaxed *Sa*FtsZ-GTP converged with the static conformation with a higher kernel density > 10 during the 200-ns MD simulation (Fig. 4b). These results suggest that the relaxed conformation of *Sa*FtsZ, regardless of bound nucleotide at the equilibrium state, is similar to the relaxed conformation previously observed. In the tense conformation, *Sa*FtsZ-GDP exhibited a conformational shift in the GAD (Fig. 3c), but the deviations with respect to the references of the relaxed and the tense conformations were almost all distributed within 1 Å and converged at a density value of 6.4 (Fig. 4c). The H9 and H10 helices of the GAD in the tense *Sa*FtsZ-GTP rotated and moved closer to the H7 helix and T7 loop (Fig. 3d), indicating that the tense *Sa*FtsZ-GTP shifted to a different conformation not previously deposited in the PDB. The conformational difference could also be explained by the finding that the trajectory was distributed far from the tense and the relaxed conformation matrices (Fig. 4d).

In *Bs*FtsZ, the average structure of the relaxed conformation bound to GDP or GTP within the last 50 ns of the MD simulation was very close to that of the reference structure of the relaxed conformation, with slight differences in some loop regions (Fig. 3e,f). The deviations in relaxed *Bs*FtsZ-GDP relative to the reference structure converged; in contrast, the conformation of relaxed *Bs*FtsZ-GTP exhibited a greater deviation relative to the reference of the tense conformation (Fig. 4e,f). In the tense conformation, a larger fluctuation was found for the T6 loop of *Bs*FtsZ-GDP. Moreover, the T10 and T11 loops in the GAD moved closer to the NTD, which was accompanied by a change in the relative orientation of the GAD (Fig. 3g). Therefore, the conformation of tense *Bs*FtsZ-GDP exhibited deviations distributed over 1.5 Å relative to the reference of the relaxed conformation (Fig. 4g). Only a small difference in the loop regions was observed in the conformation of tense *Bs*FtsZ-GTP (Fig. 3h), and consequently, the conformation converged well with the reference, with a density value of 7.2, which was two times higher than that of tense *Bs*FtsZ-GDP (Fig. 4g,h).

## Discussion

The conformations of relaxed-GDP, relaxed-GTP, and tense-GTP of both FtsZs rapidly equilibrated, with an RMSD value of < 2.0 Å relative to the initial structure (Fig. 1). On the other hand, the conformation of the tense-GDP in both FtsZs exhibited a 3.0-Å conformational change and then equilibrated later than the others, suggesting that both FtsZs share an equilibration pattern for all conformations (Fig. 1). However, the distance between the centers of the NTD and GAD in the tense *Sa*FtsZ-GDP was shifted from 28 Å observed in the tense form to 26 Å in the relaxed form (Fig. 2b). In contrast to *Sa*FtsZ, the center of the NTD of the tense *Bs*FtsZ-GDP maintained a 28-Å distance from that in the GAD (Fig. 2d), but the vector between the centers formed an angle of 5.7 ± 2.1° relative to the reference (Fig. 2h and Supplementary Table S4), suggesting that the conformation converged with the structure during the MD simulation, accompanied by a previously unreported different relative orientation between the subdomains.

Each conformation of *Bs*FtsZ exhibited less fluctuation than the corresponding conformation of *Sa*FtsZ in the last 50 ns of the simulation (Supplementary Figs. S2 and S3). *Sa*FtsZ had some fluctuating regions, such as the T6 and T7 loops connected by the H7 helix, and the T1 and T8 loops close to the H7 helix, depending on the conformational state with bound nucleotide. In particular, dynamic changes in the T1, T7, and T8 loops were specifically observed in the conformations of the tense *Sa*FtsZ. In contrast, the T3, T5, and T6 loops located near the nucleotide-binding site exhibited greater fluctuations in the tense *Bs*FtsZ. Furthermore, the T6 loop also exhibited greater flexibility in the relaxed *Ba*FtsZ-GTP and -GDP. These results suggest that *Sa*FtsZ and *Bs*FtsZ exhibit different dynamics and that the more-flexible regions are located around the H7 helix in *Sa*FtsZ and nucleotide-binding site in *Bs*FtsZ, respectively.

In the relaxed conformation, the trajectories of the conformational differences versus the reference structures suggested that the relaxed conformation fell into a narrow range of deviation relative to the reference of the relaxed structure but was widely distributed due to a larger difference relative to the reference of the tense structure (Fig. 4). Relaxed *Sa*FtsZ-GTP and relaxed *Bs*FtsZ-GDP converged with the highest densities, suggesting that FtsZ in the relaxed state exhibits a different stable condition depending on the bacterial species and bound nucleotide.

**Figure 5:**
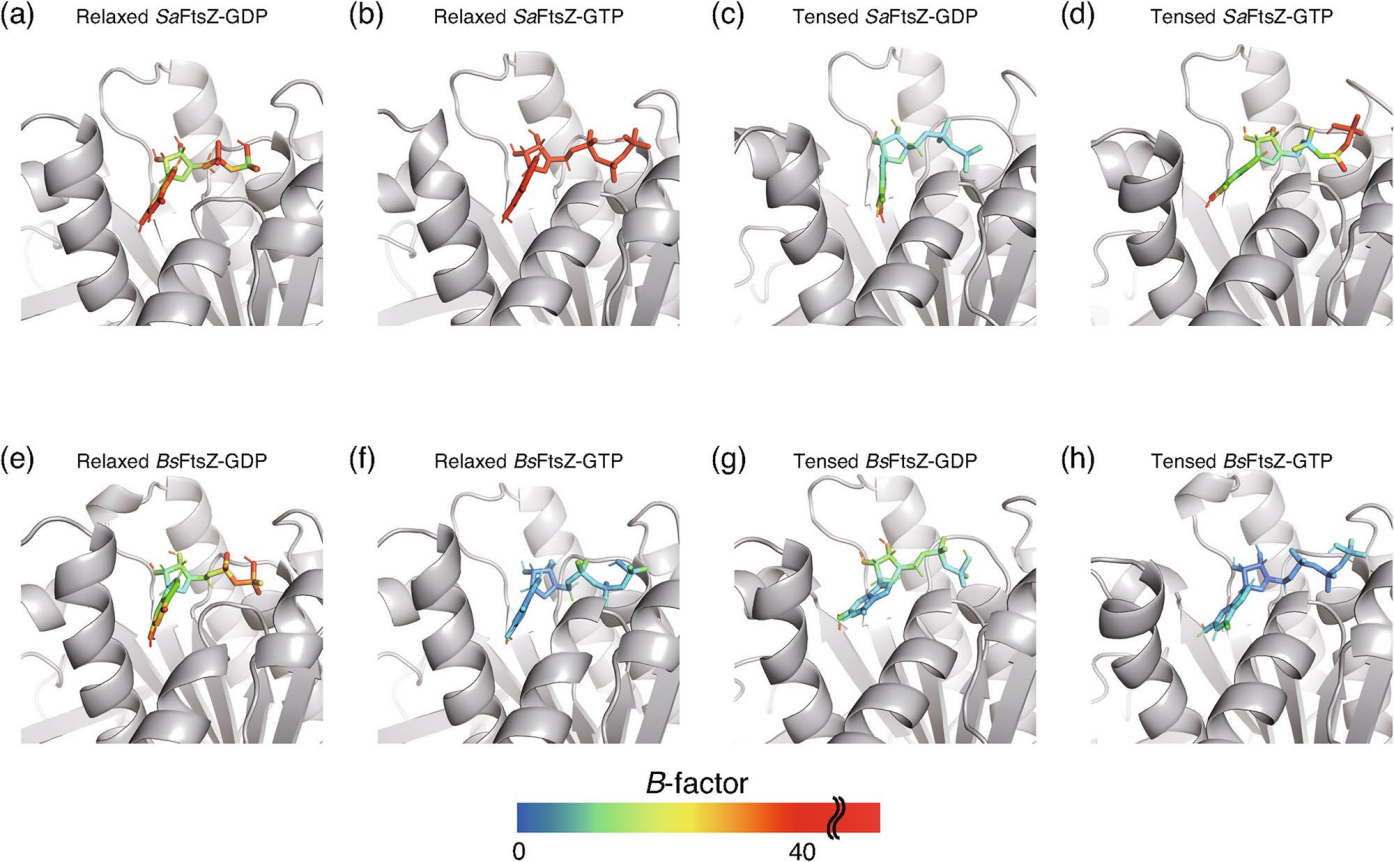
B-factor representations of atoms in the nucleotide during the last 50-ns simulation. The fluctuation behaviors of atoms in the nucleotide bound to (**a**) relaxed *Sa*FtsZ-GDP, (**b**) relaxed *Sa*FtsZ-GTP, (**c**) tense *Sa*FtsZ-GDP, (**d**) tense *Sa*FtsZ-GTP, (**e**) relaxed *Bs*FtsZ-GDP, (**f**) relaxed *Bs*FtsZ-GTP, (**g**) tense *Bs*FtsZ-GDP, and (**h**) tense *Bs*FtsZ-GTP were shown. FtsZ molecule and nucleotide were depicted by gray cartoon and sticks with the continuous scaled color of B-factor (higher; red, lower; blue).

The tense conformation exhibited different features among bacterial species. Quite marked differences were observed in the tense-GDP form. *Sa*FtsZ-GDP exhibited a converged conformation with a density value of 6.4; in contrast, *Bs*FtsZ-GDP was distributed widely and exhibited highest dynamics, with a value of < 3.15. In the tense-GTP form, however, the conformational differences of *Sa*FtsZ and *Bs*FtsZ with the reference of the relaxed conformation was approximately 2.0 and 2.5 Å with approximately 1.0-Å deviations, respectively, and *Bs*FtsZ exhibited a lower difference with the reference of the tense conformation, with a higher convergence than *Sa*FtsZ. Furthermore, the B-factors, which indicate the thermodynamical fluctuation, of atoms in the nucleotide bound to *Sa*FtsZ during the last 50 ns revealed that GTP showed higher fluctuation than GDP, and GDP bound to tense *Sa*FtsZ was more stable than others (Fig. 5a–d). In contrast to the fluctuation of the nucleotide, relaxed *Sa*FtsZ-GTP exhibited lower fluctuation than relaxed *Sa*FtsZ-GDP but tense *Sa*FtsZ-GTP was slightly more unstable than tense *Sa*FtsZ-GDP (Fig. 4), suggesting that higher fluctuation of the nucleotide influenced the lower conformational deviations in the relaxed *Sa*FtsZ but the higher conformational deviations in the tense *Sa*FtsZ conformation. However, the effect of the nucleotide stability in *Bs*FtsZ was opposite to that in *Sa*FtsZ, suggesting that the bound nucleotide affected the different stabilization in the relax- and tense-conformations of FtsZ between *Staphylococcus aureus* and *Bacillus subtilis*. These MD simulation results suggested that different features of both FtsZs affect the stability of their conformations.

X-ray crystal structures of FtsZs derived from several bacteria have been reported over the last 2 decades, when the first conformation of FtsZ was reported^4^, and all conformations reported previously were in the relaxed state before the conformation of *Sa*FtsZ was reported by independent groups^5,19,20^. The results of biochemical studies and cryoEM structural analyses of the blobbed resolution predicted that FtsZs derived from some bacteria probably assume the tense conformation^8,21, 22,35,36^, and indeed, FtsZs derived from only *Staphylococcus aureus*^5,8,11,15,16,19,20,37–39^ and *Staphylococcus epidermidis* (PDB 4M8I) gave crystal structures in the tense state^8,21,23^. The current models of treadmilling are based on the assumption that FtsZ monomers are predominantly in the relaxed conformation and that a switch to the tense conformation is caused by assembly into a protofilament^8–10^. Previous investigations also indicated that the filament may promote not only the stability of the tense conformation^8,21^ but also the transition to the relaxed conformation^23^. The results of MD simulations in this study suggested distinct dynamic properties of monomeric *Sa*FtsZ and *Bs*FtsZ. The relaxed *Bs*FtsZ-GDP was identified as the most stable conformation among all conformations of *Bs*FtsZ in the solution, consistent with the models proposed by the treadmilling^8–10^. On the other hand, the tense *Sa*FtsZ-GDP surprisingly exhibited more stability than the relaxed *Sa*FtsZ-GDP. The monomer structure of the relaxed *Sa*FtsZ in the MD simulation was generated from the relaxed conformations of *Sa*FtsZ longitudinally assembled in the crystal. The influence of the longitudinal interaction likely persisted, especially as only the T7 loop in the relaxed *Sa*FtsZ adopts a markedly distinct conformation^16^. This caused the relaxed *Sa*FtsZ model to settle into a higher energy state, conceivably a local minimum, compared to the monomeric state, despite undergoing energy minimization and equilibration. To realize a stable conformation of the relaxed *Sa*FtsZ as calculated by the MD simulation, it is presumed that extending the time of an equilibration MD run or the crystal structure of the relaxed *Sa*FtsZ with the conserved conformation of the T7 loop as a monomer state could be necessary.

MD simulations also suggested that the monomeric tense FtsZ derived from both bacteria did not spontaneously show the conformational transition from the tense to the relaxed conformations in the solution. Therefore, the transition to the relaxed conformation may be regulated by the protofilament instead of the spontaneous conformational change in the solution. By resolving the crystal structure of the relaxed *Sa*FtsZ at the monomeric state and the detailed mechanisms in which residues or regions contribute to the stability of these conformations in the future, a greater understanding of the machinery that regulates conformational changes and filament treadmilling will be deeply understood.

## Methods

### MD simulations

MD simulations were performed using GROMACS 2016.3^40^, as described in previous studies^41,42^. *Sa*FtsZ from 5H5G molecules A and B^16^ and *Bs*FtsZ from 2VXY molecule A^14^ were used for this study. The model structure of *Bs*FtsZ was built with SWISS-MODEL^32^ using PDB 3VOA derived from *Sa*FtsZ^5^ as a template. The metal ion coordinated in the PDB was removed as followed by previous MD simulation^23^. For each simulated system, the FtsZ molecule was also placed at the center of a rectangular periodic boundary box filled with water molecules. The size of the box used for the FtsZ molecule is summarized in Supplementary Table 1. The protonation states of residues and the hydrogen coordinates of FtsZ were determined using MOE (MOLSIS Inc., Tokyo, Japan)^24^. For simulation of the FtsZ-GTP complex, the GTP molecule was superposed with GDP bound to FtsZ, and the coordinates of the GDP molecule were replaced with those of GTP. The N- and C-termini of the FtsZ molecules were capped with an acetyl group and *N*-methyl group, respectively, using MOE 2020.09 (MOLSIS Inc.). Instead of *N*-methyl capping, a negative point charge was added to the C-terminus of FtsZ derived from PDB 5H5G molecule A. Sodium ions were added to neutralize the entire system as followed by the previous MD simulation of *Mtb*FtsZ^23^. AMBER ff99SB-ILDN energy parameters^43^ were used for FtsZ and the sodium ion, and the TIP4P-Ew model^44^ was used for water molecules. The general Amber force field^45^ was used for GDP and GTP. The partial atomic charges of GDP and GTP were calculated using the restrained electrostatic potential^46^ methodology based on DFT calculations (B3LYP/6-31G*) using the Gaussian 09 revision E01 program package (Gaussian, Inc., Wallingford CT, 2016)^47^. After running the steepest descent energy minimization, relaxations were run at 250 K and 310 K for 1 ns each, with positional restraints of 1000 kJ mol^−1^ nm^−2^ for all non-hydrogen atoms of FtsZ and the nucleotides. Subsequently, the system was pre-equilibrated at 310 K for 1 ns and 1 bar using a Berendsen barostat^48^. An equilibration MD simulation without positional restraints was performed at 310 K and 1 bar for 200 ns. The temperature and pressure were maintained using a velocity rescaling thermostat and Parrinello-Rahman barostat with relaxation times of 0.2 and 5.0 ps, respectively^41^. All bonds connected to hydrogen atoms were constrained using the LINCS^49^ algorithm, and the time step was set to 2 fs. Long-range Coulomb interactions were calculated using the smooth particle mesh Ewald^50^ method, with a grid spacing of 0.30 nm. The real space cut-off for both the Coulomb and van der Waals interactions was 1.2 nm.

The geometric center of NTD and GAD were obtained from the average coordinates of all Cα atoms in the range of residue 12–173 and 223–310, respectively (Supplementary Fig. S6). The inter-subdomain distance between the geometric centers of each subdomain was calculated each time step. The vector angle was calculated from the dot product between the inter-subdomain vector at each step and the vector at 0 ns (Supplementary Fig. S7). The thermodynamical fluctuation of the atoms in the nucleotide while the last 50-ns simulation was calculated as B-factor^51^.

## Supplementary Information

Supplementary Information.

Supplementary Video 1.

Supplementary Video 2.

Supplementary Video 3.

Supplementary Video 4.

Supplementary Video 5.

Supplementary Video 6.

Supplementary Video 7.

Supplementary Video 8.

## Data Availability

The raw data generated in this study have been deposited in the Zenodo 10.5281/zenodo.10881059 and available from the corresponding authors upon request.
