# Supplementary material for: Molecular dynamics simulations reveal differences in the conformational stability of FtsZs derived from Staphylococcus aureus and Bacillus subtilis
Source: Sci Rep. 2024 Jul 11;14:16043. doi: 10.1038/s41598-024-66763-x (PMC11239868; doi:10.1038/s41598-024-66763-x)
Supplement: Supplementary file 1 — Supplementary Information. [file 41598_2024_66763_MOESM1_ESM.pdf]

Supplementary materials for

Molecular dynamics simulations reveal differences in the conformational stability of FtsZs derived from *Staphylococcus aureus* and *Bacillus subtilis*

Taichi Takasawa[1], Takashi Matsui[1,2,*], Go Watanabe[3,4,*], and Yoshio Kodera[1,4]

[1] Department of Physics School of Science, Kitasato University, 252-0373, Japan

[2] Center for Disease Proteomics, School of Science, Kitasato University, 252-0373, Japan

[3] Department of Data Science, School of Frontier Engineering, Kitasato University, 252-0373, Japan

[4] Kanagawa Institute of Industrial Science and Technology (KISTEC), 243-0435, Japan

*Correspondence: Dr. Takashi Matsui, e-mail: matsui@kitasato-u.ac.jp, Postal address: 1-15-1 Kitasato, Minami-ku, Sagamihara, Kanagawa 252-0373, Japan. Prof. Go Watanabe, e-mail: go0325@kitasato-u.ac.jp, Postal address: 1-15-1 Kitasato, Minami-ku, Sagamihara, Kanagawa 252-0373, Japan

Table of contents

Supplementary Tables and Figures.

Table S1.	Size of boxes used for MD simulations.
Table S2.	Inter-subdomain distances of reference structures.
Table S3	Inter-subdomain angles of simulation structures derived from *Sa*FtsZ, with the reference structures.
Table S4.	Inter-subdomain angles of simulation structures derided from BsFtsZ, with the reference structures.
Figure S1.	Conformation of SaFtsZ and superimposing of Relaxed BsFtsZ with Relaxed and Tensed SaFtsZ.
Figure S2.	RMSF values mapped on the structures of *Sa*FtsZ.
Figure S3.	RMSF values mapped on the structures of *Bs*FtsZ.
Figure S4.	Superimposing the average structure during the last 50 ns of MD simulations, with the GAD of the crystal structure.
Figure S5.	The inter-subdomain distance.
Figure S6.	The inter-subdomain vector angles.
Figure S7.	Average structure during the last 50 ns of MD simulations, compared with the crystal structure.
Movie S1.	Relaxed *Sa*FtsZ GDP
Movie S2.	Relaxed *Sa*FtsZ GTP
Movie S3.	Tense *Sa*FtsZ GDP
Movie S4.	Tense *Sa*FtsZ GTP
Movie S5.	Relaxed *Bs*FtsZ GDP
Movie S6.	Relaxed *Bs*FtsZ GTP
Movie S7.	Tense *Bs*FtsZ GDP
Movie S8.	Tense *Bs*FtsZ GTP

Supplementary Table S1. Size of boxes used for MD simulations.

		Cell size (nm^3)	No of water molecules	No. of sodium ions
*Sa*FtsZ	Relax-GDP	10 × 9 × 10	28,319	18
	Relax-GTP	9 × 9 × 9	22,802	19
	Tense-GDP	10 × 10 × 10	31,565	20
	Tense-GTP	10 × 10 × 10	31,564	21
*Bs*FtsZ	Relax-GDP	10 × 9 × 10	28,349	13
	Relax-GTP	10 × 9 × 10	28.348	14
	Tense-GDP	10 × 10 × 10	31,573	13
	Tense-GTP	10 × 10 × 10	31,571	14

Supplementary Table S2. Inter-subdomain distances of reference structures.

Reference structure (Relax-form)						
Organism	*Sa*	*Bs*	*Kp*	*Ec*	*Mj*	Average
PDB ID	5H5G mol B	2VXY	6LL5	6LL6	1W59 mol A	
Intersubdomain Distance (Å)	26.6	25.7	26.8	26.1	25.7	26.2±0.5

Reference structure (Tense-form)				
Organism	*Sa*		*Se*	Average
PDB ID	5H5G mol A	3VOA	4M8I	
Intersubdomain Distance (Å)	28.3	28.4	28.4	28.4±0.1

Sa; *Staphylococcus aureus*, *Bs*; *Bacillus subtilis*, *Kp*; *Klebsiella pneumoniae*, *Ec*; *Escherichia coli*, *Mj*; *Methanocaldococcus jannaschii*, *Se*; *Staphylococcus epidermidis*.

Supplementary Table S3. Inter-subdomain angles of simulation structures derived from *Sa*FtsZ, with the reference structures.

Reference structure (Relax-form)					
Organism	*Sa*	*Bs*	*Kp*	*Ec*	*Mj*
PDB ID	5H5G mol B	2VXY	6LL5	6LL6	1W59 mol A
Relax-form GDP	6.8°±1.8°	2.0°±1.0°	7.9°±1.8°	5.7°±1.8°	2.1°±1.0°
Relax-form GTP	2.8°±1.1°	5.1°±1.1°	6.9°±1.2°	4.2°±1.2°	4.5°±1.1°
Tense-form GDP	4.7°±1.6°	10.9°±2.1°	5.8°±2.2°	5.7°±2.1°	11.0°±1.9°
Tense-form GTP	7.4°±1.4°	12.1°±2.0°	5.8°±2.6°	6.9°±2.0°	12.5°±1.8°

Reference structure (Tense-form)			
Organism	*Sa*		*Se*
PDB ID	5H5G mol A	3VOA	4M8I
Relax-form GDP	10.9°±1.9°	10.0°±1.9°	9.5°±1.9°
Relax-form GTP	7.3°±1.1°	6.6°±1.2°	6.3°±1.2°
Tense-form GDP	2.7°±1.6°	3.0°±1.5°	3.3°±1.5°
Tense-form GTP	3.6°±1.6°	3.8°±1.6°	3.9°±1.7°

Sa; *Staphylococcus aureus*, *Bs*; *Bacillus subtilis*, *Kp*; *Klebsiella pneumoniae*, *Ec*; *Escherichia coli*, *Mj*; *Methanocaldococcus jannaschii*, *Se*; *Staphylococcus epidermidis*.

Supplementary Table S4. Inter-subdomain angles of simulation structures derived from *Bs*FtsZ, with the reference structures.

Reference structure (Relax-form)					
Organism	*Sa*	*Bs*	*Kp*	*Ec*	*Mj*
PDB ID	5H5G mol B	2VXY	6LL5	6LL6	1W59 mol A
Relax-form GDP	6.0°±1.3°	2.0°±1.0°	7.7°±1.3°	5.4°±1.4°	1.9°±1.0°
Relax-form GTP	5.2°±1.4°	2.4°±1.2°	6.8°±1.1°	4.5°±1.2°	2.5°±1.1°
Tense-form GDP	9.9°±1.9°	12.2°±1.4°	4.8°±1.4°	7.3°±1.5°	13.4°±1.4°
Tense-form GTP	6.0°±1.3°	10.9°±1.5°	5.2°±1.5°	5.6°±1.5°	11.5°±1.4°

Reference structure (Tense-form)			
Organism	*Sa*		*Se*
PDB ID	5H5G mol A	3VOA	4M8I
Relax-form GDP	9.7°±1.4°	8.9°±1.4°	8.9°±1.4°
Relax-form GTP	9.0°±1.3°	8.1°±1.3°	7.6°±1.3°
Tense-form GDP	6.7°±2.3°	6.1°±2.2°	5.7°±2.1°
Tense-form GTP	2.1°±1.2°	2.1°±1.1°	2.2°±1.2°

Sa; *Staphylococcus aureus*, *Bs*; *Bacillus subtilis*, *Kp*; *Klebsiella pneumoniae*, *Ec*; *Escherichia coli*, *Mj*; *Methanocaldococcus jannaschii*, *Se*; *Staphylococcus epidermidis*.

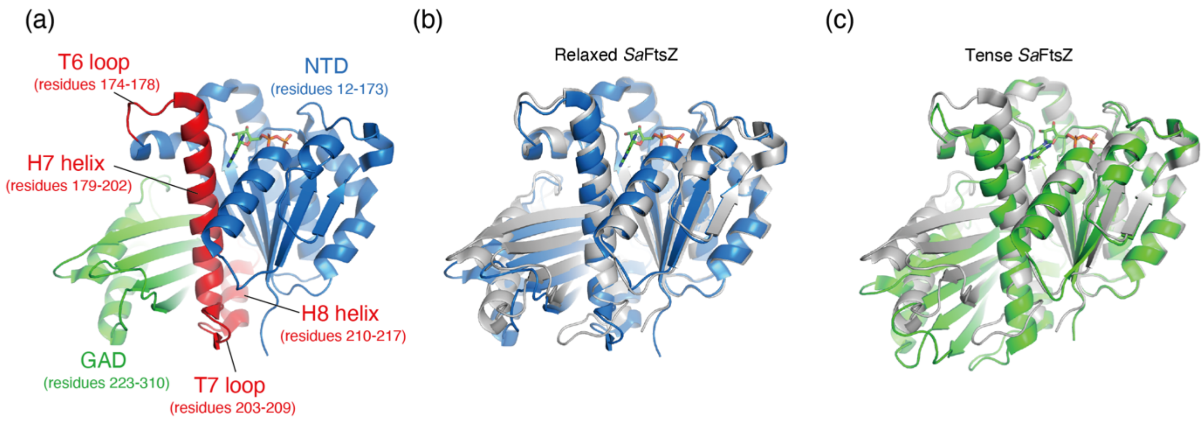

Supplementary Figure S1. Conformation of *Sa*FtsZ and superimposing of relaxed *Bs*FtsZ with relaxed and tense *Sa*FtsZ. The cartoon model of relaxed SaFtsZ (PDB 5H5G, model B). NTD, GAD, and the linker region flanked by NTD and GAD were depicted as blue, green, and red cartoons, respectively. (b, c) Superimposing the NTD of the relaxed *Bs*FtsZ (PDB 2VXY, gray) with those of (b) relaxed (PDB 5H5G, model B, blue) and (c) tense *Sa*FtsZ (PDB 5H5G, model A, green).

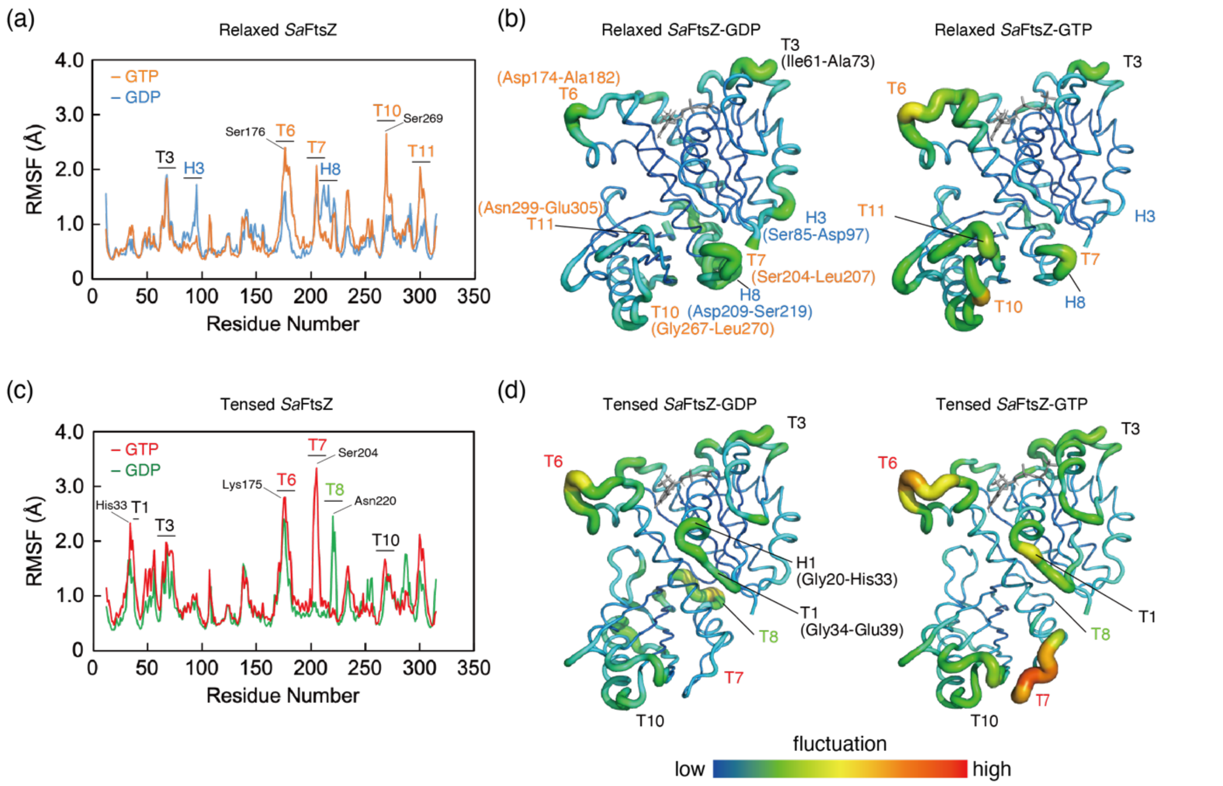

Supplementary Figure S2. RMSF values mapped on the structures of *Sa*FtsZ. (a, b) RMSF values of (a) relaxed and (b) tense *Sa*FtsZ across residues 12-316 during the last 50 ns of MD simulations. (a) Relaxed conformation bound to GTP and GDP drawn as orange and blue lines, respectively. (b) Tense conformation bound to GTP and GDP depicted as red and green lines, respectively. (c, d) RMSF values mapped onto the crystal structures (upper; bound to GDP, lower; bound to GTP). RMSF values indicated in panels (a) and (b) mapped onto the crystal structure (PDB 5H5G, mol B, and A), respectively. Differences in RMSF values between binding of GTP and GDP are indicated in each panel. The fluctuation is shown as continuous scaled color (higher; red, lower; blue).

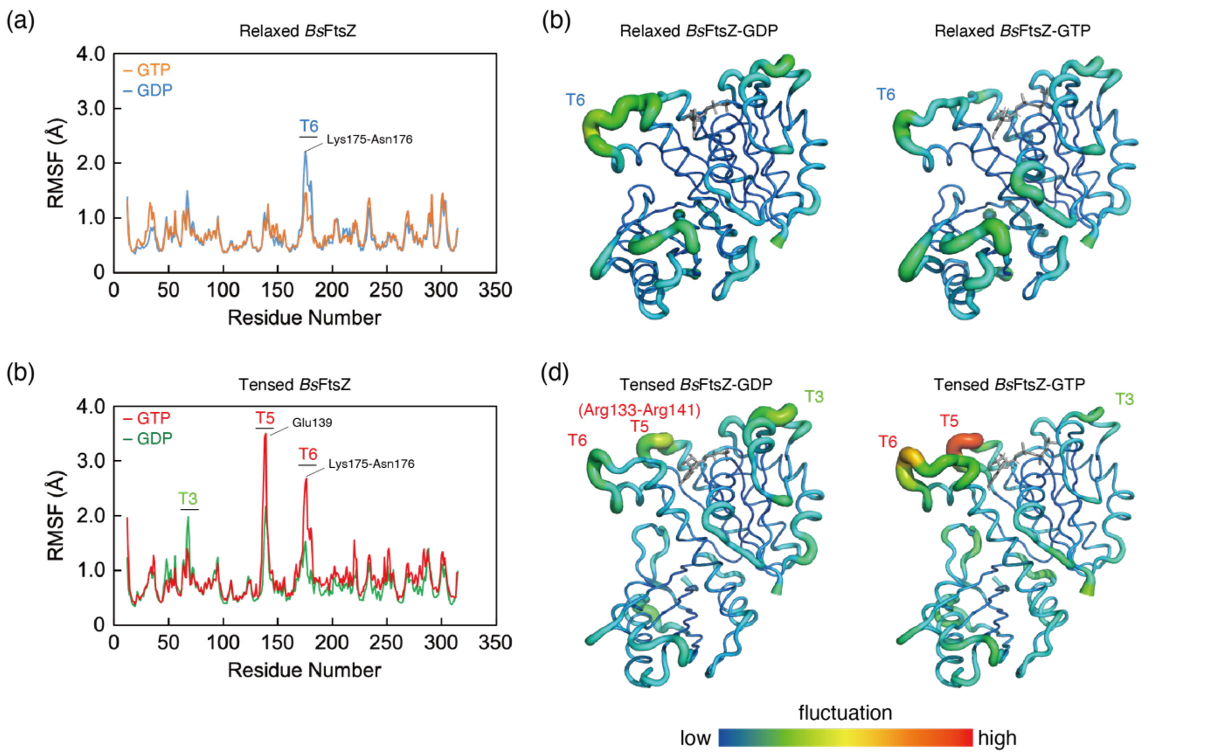

Supplementary Figure S3. RMSF values mapped onto the structures of *Bs*FtsZ. (a, b) RMSF values of (a) relaxed and (b) tense *Bs*FtsZ across residues 12-316 during the last 50 ns of MD simulations. (a) Relaxed conformation bound to GTP and GDP drawn as orange and blue lines, respectively. (b) Tense conformation bound to GTP and GDP depicted as red and green lines, respectively. (c, d) RMSF values mapped onto the crystal structure (upper; bound to GDP, lower; bound to GTP). RMSF values indicated in panels (a) and (b) mapped onto the crystal structure (PDB 5H5G, mol B, and A), respectively. Differences in RMSF values between binding of GTP and GDP are indicated in each panel. The fluctuation is shown as continuous scaled color (higher; red, lower; blue).

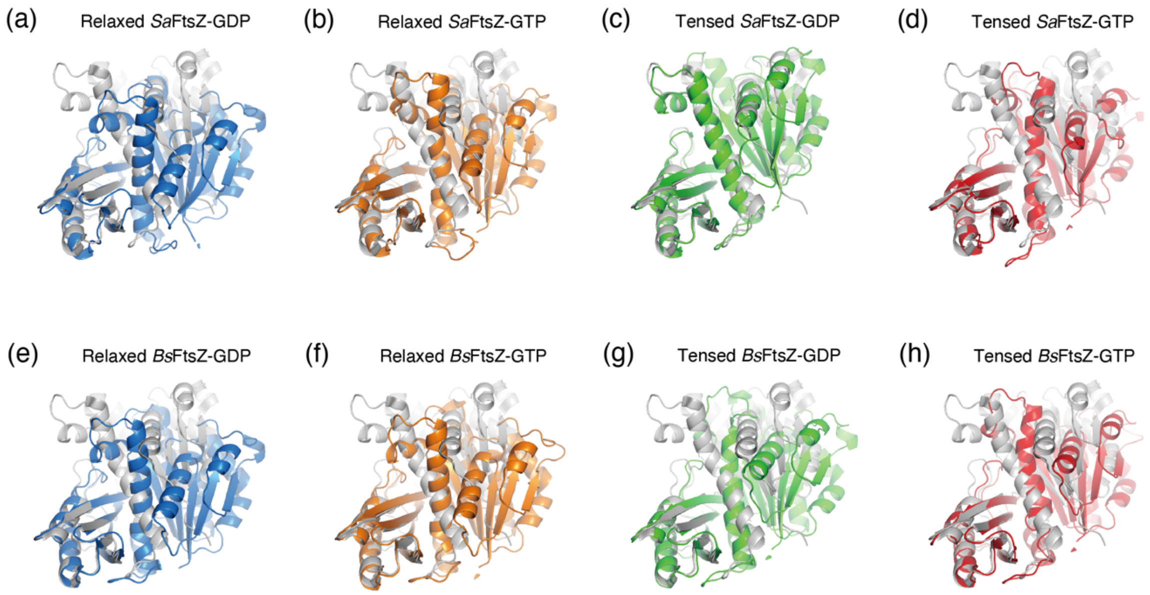

Supplementary Figure S4. Superimposing the average structure during the last 50 ns of MD simulations, with the GAD of the crystal structure. GAD of the average structures of (a) relaxed *Sa*FtsZ-GDP, (b) relaxed *Sa*FtsZ-GTP, (c) tense *Sa*FtsZ-GDP, (d) tense *Sa*FtsZ-GTP, (e) relaxed *Bs*FtsZ-GDP, (f) relaxed *Bs*FtsZ-GTP, (g) tense *Bs*FtsZ-GDP, and (h) tensed *Bs*FtsZ-GTP were superimposed with that of crystal structure of *Sa*FtsZ (PDB 3VOA).

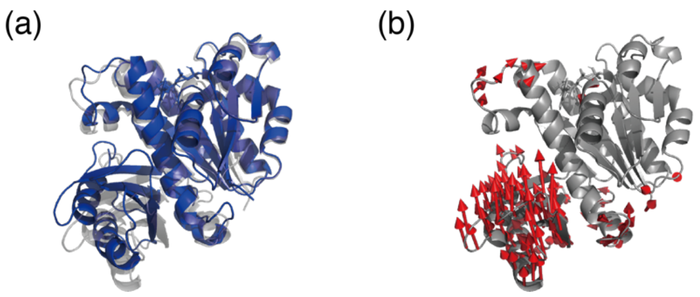

Supplementary Figure S5. Average structure during the last 50 ns of MD simulations, compared with the crystal structure. (a) Average structure of the relaxed form bound to GDP during the MD simulation compared with the crystal structure (PDB 5H5G mol B[12]). (b) Conformational changes from the crystal structure to the average structure indicated in panel (a) are illustrated as vectors. Coordinate differences >2.0 Å are indicated by red arrows.

(a) (b)

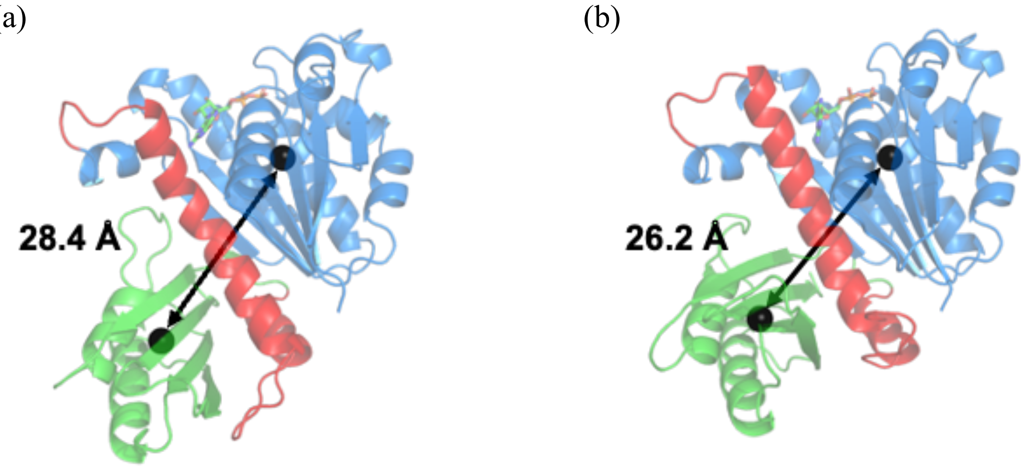

Supplementary Figure S6. The inter-subdomain distance. (a, b) The inter-subdomain distances between each subdomain at (a) the tense conformation (PDB 3VOA) and (b) the relaxed conformation (PDB 5H5G, model B) were calculated as the value of 28.4 and 26.2 Å, respectively. This figure is displayed in the same manner as in Supplementary Figure S1.

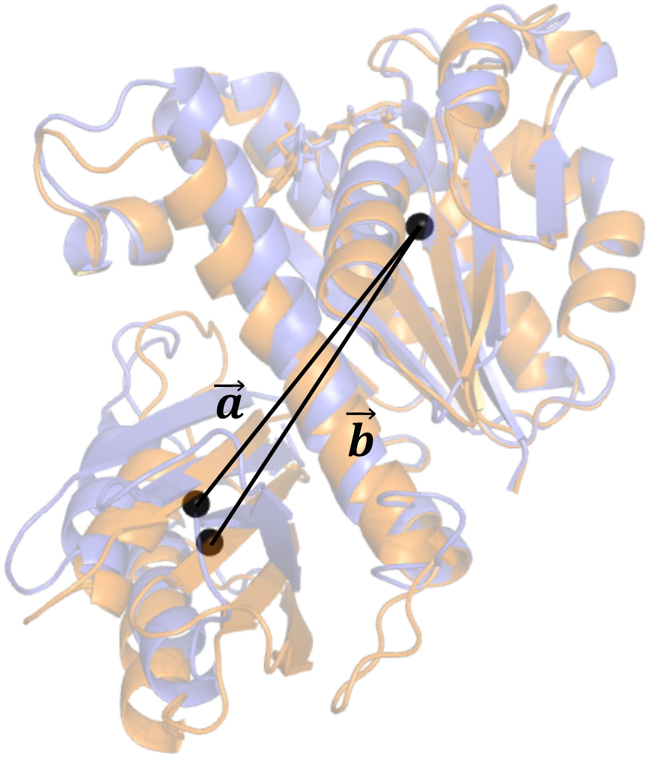

Supplementary Figure S7. The inter-subdomain vector angles. The inter-subdomain angles between the inter subdomain vectors at each simulation steps (blue cartoon, $\vec{a}$) and the vector at 0 ns (orange cartoon, $\vec{b}$) were calculated. The inter subdomain vector was derived from the calculation of the inter-subdomain distance at each simulation step.
